# Composition of a Salivary Calculus Taken from a Horse

**Published:** 1848-10

**Authors:** 


*Composition of a Salivary Calculus taken from a Horse,* by J. L. Lassigne.—The calculus was removed from the neck of the first molar tooth of the left upper jaw. In less than a year it had attained the size of a hen's egg: it was of a reddish-white color, remarkably hard, and of a laminated structure. It dissolved in dilute nitric acid with effervescence, and deposited some mucous flocculi. The filtered solution, saturated with ammonia, threw down a white precipitate of phosphate of lime. Analysis of a portion of the calculus furnished the following results:—

| | |
|---|---|
| Water | 3.27 |
| Soluble salivary matter | 6.19 |
| Mucus | 4.50 |
| Phosphate of lime | 2.70 |
| Carbonate of lime | 83.36 |

The essential ingredient in the calculus, therefore, was carbonate of lime, whilst in human salivary calculi the principal ingredient is phosphate of lime.—*Helleir's Archiv. Sept.* 2, 1846.

